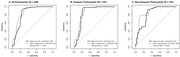# Plasma Aβ42/40, *APOE4*, and Ethnicity in Aβ‐PET Prediction

**DOI:** 10.1002/alz.095051

**Published:** 2025-01-09

**Authors:** Steven W Taylor, Darren M Weber, Robert J Lagier, Matthew A Stroh, Nigel J Clarke, David Vaillancourt, Ranjan Duara, Michael K Racke

**Affiliations:** ^1^ Quest Diagnostics Nichols Institute, San Juan Capistrano, CA USA; ^2^ 1Florida Alzheimer’s Disease Research Center, Gainesville, FL USA; ^3^ Wien Center for Alzheimer’s Disease Research Center, Miami Beach, FL USA; ^4^ Mount Sinai Medical Center, Miami Beach, FL USA; ^5^ 1Florida Alzheimer’s Disease Research Center, Miami, FL USA

## Abstract

**Background:**

Addition of apolipoprotein E4 (ApoE4) proteotype (as a surrogate for high‐risk *APOE* ɛ4 genotype) has significantly improved models that use the plasma Aβ42/40 ratio to predict Aβ PET‐positivity, but studies suggest different ethnicities may be less affected by the addition. Our objective was to assess the influence ApoE4 proteotype in a predominantly Hispanic cohort of participants.

**Method:**

Participants in this study (n = 250) underwent neurological and neuropsychological evaluations and amyloid PET brain scans (SUVR data and visual reads) at the 1Florida ADRC; they also underwent blood draws for ApoE4 proteotyping and Aβ42/40 ratio determination by LC‐MS/MS performed at Quest Diagnostics. Ethnicity was self‐reported as Hispanic or non‐Hispanic. Using logistic regression analysis and Delong’s test, we examined the effect of incorporating ApoE4 proteotype as a categorical variable on AUCs for the overall population and for participants stratified by ethnicity: Hispanic vs non‐Hispanic. Statistical analyses were conducted using R statistical software version 4.3.3. Two‐tailed P‐values <0.05 were considered statistically significant.

**Result:**

The ADRC cohort had roughly 3‐times the percentage of Hispanic participants (about 60% of both the Aβ PET‐positive and Aβ PET‐negative participants) compared with a previous Aβ42/40 LC‐MS/MS study that incorporated ApoE4 proteotype. Based on ApoE4 proteotype, incorporating *APOE4* allele count modestly but significantly increased the AUC (95% CI) from 0.84 (0.79‐0.89) to 0.86 (0.82‐0.91), *P* = 0.038, **Figure A**. Subsetting the ADRC cohort participants into Hispanic vs non‐Hispanic ethnicity provided a contrasting effect for ApoE4 proteotype on model improvement. For Hispanic participants, the AUC (95% CI) for the ratio plus ApoE4 proteotype, 0.86 (0.80 to 0.92) and the Aβ42/40 ratio alone, 0.85 (0.78‐0.91) were not significantly different (*P* = 0.306), **Figure B**. For non‐Hispanic participants, the AUC (95% CI) for the ratio plus ApoE4 proteotype, 0.87 (0.81‐0.94) was significantly higher (*P* = .048) than the Aβ42/40 ratio alone, 0.83 (0.75‐ 0.91), Figure C.

**Conclusion:**

ApoE4 proteotype did not improve prediction of PET positivity by plasma Aβ42/40 in the Hispanic subcohort, suggesting that adding *APOE4* to Aβ42/40 in algorithms used in Alzheimer’s disease risk assessment may not improve PET‐prediction for all races and ethnicities.